# The Effect of a Consumer-Based Activity Tracker Intervention on Accelerometer-Measured Sedentary Time Among Retirees: A Randomized Controlled REACT Trial

**DOI:** 10.1093/gerona/glab107

**Published:** 2021-04-11

**Authors:** Kristin Suorsa, Tuija Leskinen, Anna Pulakka, Jaana Pentti, Eliisa Löyttyniemi, Ilkka Heinonen, Jussi Vahtera, Sari Stenholm

**Affiliations:** 1 Department of Public Health, University of Turku and Turku University Hospital, Finland; 2 Centre for Population Health Research, University of Turku and Turku University Hospital, Finland; 3 Finnish Institute for Health and Welfare, Helsinki, Finland; 4 Department of Biostatistics, University of Turku and Turku University Hospital, Finland; 5 Turku PET Centre, and Department of Clinical Physiology and Nuclear Medicine, University of Turku, Turku, Finland; 6 Rydberg Laboratory of Applied Sciences, Department of Environmental and Biosciences, University of Halmstad, Sweden

**Keywords:** Prolonged sedentary time, Prompt, Retirement, Self-monitoring, Wearable device

## Abstract

**Background:**

Effective strategies to reverse the increasing trend of sedentary behavior after retirement are needed. The aim of this study was to examine the effect of 12-month activity tracker-based intervention on daily total and prolonged sedentary time (≥60 minutes) among recent retirees.

**Methods:**

Randomization to intervention and control groups was performed to 231 retirees (mean age 65.2 [*SD* 1.1] years, 83% women). Intervention participants wore a consumer-based wrist-worn activity tracker (Polar Loop 2, Polar, Kempele, Finland), including daily activity goal, every day and night for 12 months. The activity tracker also gave vibrating reminders to break up uninterrupted inactivity periods after 55 minutes. A wrist-worn triaxial ActiGraph wGT3X-BT accelerometer was used to measure sedentary time at baseline and at 3-, 6-, and 12-month time points.

**Results:**

The use of an activity tracker did not reduce daily total or prolonged sedentary time over 12 months (*p* values for time * group interaction 0.39 and 0.27, respectively). In the post hoc analysis focusing on short- and medium-term effects on prolonged sedentary time, no differences between the intervention and control groups over 3 months were found, but a tendency for a greater decrease in prolonged sedentary time in the intervention group over 6 months was seen (mean difference in changes between the groups 29 minutes, 95% CI −2 to 61).

**Conclusions:**

The activity tracker with inactivity alerts did not elicit changes in sedentary time over 12 months among recent retirees. Alternative approaches may be needed to achieve long-term changes in sedentary time among retirees.

**Clinical Trials registration Number:** NCT03320746

## Background

Sedentary time covers a large portion of daily waking hours among adults aged 60 or older in high-income countries (1). A compelling evidence associates sedentary time with compromised cardiometabolic health, lower physical function and higher mortality (2,3). High daily sedentary time has been observed to be especially harmful in relation to glucose regulation, cardiovascular health, and mortality, when it is accumulated from long uninterrupted bouts (4–7). Furthermore, sedentary time seems to increase with advancing age (8), highlighting the need for preventive actions to support healthy aging.

New ways to reduce sedentary time are particularly needed among recent retirees, because retirement induces unfavorable changes in sedentary behavior. Recent accelerometer-based findings have indicated increases in daily total and prolonged sedentary time in the transition to retirement (9,10), and survey studies have shown that mainly time spent watching TV increases (11,12). Watching TV may be more harmful for health compared to other sedentary activities, because sitting while watching TV is likely more prolonged (13). However, no interventions to reduce and break up sedentary time have been targeted to the time window immediately after transition to retirement (14), which could be a potential time point to attenuate or reverse the increasing trend of sedentary behavior in this age group.

Compared to traditional face-to-face and counseling interventions, wearable technology, such as activity trackers, may offer a less resource-intensive and more scalable, practical and personalized method to deliver multiple behavioral change techniques (BCTs) (15), such as self-monitoring and feedback, and to promote changes in activity behavior in large populations (16). Combining activity tracker with counseling and educational sessions has provided promising results in reducing sedentary time among middle-aged and older adults (17–19). However, only a few randomized controlled trials (RCTs) have evaluated the effect of an activity tracker as the primary intervention component on sedentary time, and these are conducted solely among young and middle-aged adults (20–22). In these RCTs, the main BCT has been self-monitoring which according to the Social Cognitive Theory may help to bring habitual behavior, such as sedentary behavior, into conscious awareness and to promote self-control (23–25). An Australian 1-month-long activity tracker intervention utilized self-monitoring of both physical activity and sedentary behavior and resulted in about 2 hours greater reduction in self-reported daily sedentary time than among the controls (*n* = 33, mean age 36) (21). Two other interventions utilized only self-monitoring of physical activity, which turned out to be an insufficient method to either increase physical activity or to reduce sedentary time among healthy Finnish men (*n* = 276, mean age 18) over 3 months (21), and Singaporean workers (*n* = 800, mean age 36) over 6 months (22). Thus, further studies are warranted to examine the long-term effect of an activity tracker that enables self-monitoring of sedentary time.

Sedentary behavior is habitual by its nature, meaning that it often takes place without specific intentions or reasoning (26). One promising BCT to target habitual sedentary behavior is prompts, which can act as essential external signals to trigger the recommended behavior among people convinced of the health benefits of reducing sedentary behavior (27). Recent systematic reviews suggest that computer- or phone-delivered reminders delivered are promising techniques to reduce sedentary time, but the current evidence relies on short-term interventions conducted mainly at workplaces (28,29). Among the UK office workers (*n* = 28, aged 40–50 years), computer software that reminded the user to break up occupational sitting every 30 minutes reduced worktime prolonged sedentary time (>30 minutes) by 1 hour more among the intervention group than among the controls during a usual workweek (30). However, the effect of prompts on nonoccupational sedentary behavior needs to be elucidated.

The greatest health benefits may be achieved when daily sedentary time is limited and prolonged sedentary time is broken up with either light physical activity or moderate-to-vigorous physical activity, and moderate-to-vigorous physical activity is performed at least 150 minutes per week (31). Based on cross-sectional studies, reallocating 30 minutes of sedentary time to light physical activity or moderate-to-vigorous physical activity is associated with clinically meaningful improvements in waist circumference, fasting insulin, and high-density lipoprotein cholesterol (32). Therefore, it is justifiable to target all modes of activity by utilizing different BCTs. In general, physical activity interventions and combined physical activity and sedentary behavior interventions have been less effective in reducing sedentary time when compared with sedentary behavior-focused interventions (23,33). One explanation may be that most of the physical activity and combination interventions have attempted to reduce sedentary time by exercise programs and self-monitoring of physical activity by pedometers (23,33), whereas sedentary behavior interventions have utilized self-monitoring of sedentary behavior (23) and adjustable workstations in office settings (33). Therefore, physical activity interventions may be more effective in reducing sedentary time if they also include salient BCTs to target sedentary behavior.

The REACT trial was designed to examine the effect of a 12-month-long consumer-based activity tracker intervention primarily on physical activity and secondarily on sedentary time among recent retirees. The activity tracker used as the intervention tool included self-management strategies targeted to both physical activity and sedentary behavior. The results for the primary outcome of the trial are reported elsewhere (34). This study describes the effect of a consumer-based activity tracker incorporated with inactivity alerts, on accelerometer-measured daily total and prolonged sedentary time. It was hypothesized that the activity tracker’s inactivity alerts will provoke users to break up long uninterrupted inactivity periods, thus reducing mainly prolonged sedentary time and also daily total sedentary time. Secondly, it was hypothesized that the activity tracker will encourage users to increase their daily total physical activity, leading to reduction in daily total sedentary time.

## Method

### Trial Design

This study is based on the enhancing physical activity and healthy aging among recent retirees (REACT) RCT, conducted at the University of Turku, Finland in 2018–2020. The aim of the REACT trial was to investigate the effect of an activity tracker-based intervention on physical activity as a primary outcome and sedentary behavior as well as sleep as secondary outcomes, in comparison to a control group receiving no intervention.

The REACT trial was conducted according to the guidelines of good scientific practice set by the National Advisory Board on Research Ethics in Finland and the Declaration of Helsinki. The REACT trial (registration number NCT03320746) was approved by the ethics committee of Hospital District of Southwest Finland (107/1801/2017) and all participants provided a signed, informed consent before the randomization to intervention and control groups. The data were collected and analyzed according to CONSORT guidelines (flow diagram as Supplementary File 1, checklist as the Supplementary File 2).

### Recruitment and Participants

The target population for the REACT trial consisted of Finnish public sector employees whose estimated individual statutory retirement date was between January 2016 and April 2019 and who lived in Southwest Finland in 2017 (*n* = 1475). Information on the estimated individual statutory retirement date was obtained from Keva, the pension insurance institute for the municipal sector in Finland. The target population of the REACT trial first contacted in January 2018, by mailing them an invitation letter including information on the REACT trial and inclusion criteria. The enrollment continued to March 2018. The inclusion criteria were actual retirement date between January 2016 and December 2018, self-reported ability to walk 500 m without interruptions, no current postoperative state or no known surgery within 6 months, no malignant cancer or recent myocardial infarction, basic knowledge of how to use a computer, and internet access at home. Overall, 272 individuals (18% of the target population) expressed an interest in taking part in the trial. The proportion of women and the highly educated was higher among the respondents than among the nonrespondents (82% vs 78%, 37% vs 20%, respectively). Of the respondents, 252 fulfilled the inclusion criteria and were invited to the baseline clinical and accelerometer measurements.

Eventually, 231 of the invited 252 individuals were able to participate in the baseline clinical and accelerometer measurements (Supplementary File 1) conducted by the study nurse and researcher responsible for the delivery of the intervention. After the baseline measurements, a statistician not involved in the running of the REACT trial randomized these 231 participants into the intervention and the control groups, stratified by gender, with an allocation ratio of 1:1 using a random permuted block method with SAS software.

### Intervention

The intervention was delivered by a consumer-based activity tracker (Polar Loop 2, Polar, Kempele, Finland), which was posted with its instructions to the intervention group participants. The intervention group members were requested to wear the activity tracker on their nondominant wrist at all times day and night, over a 12-month period and to download their activity data to manufacturer’s web-based program, Polar Flow (35), once a week. The participants gave the researchers a permission to follow the use of the trackers in the web-based program throughout the intervention.

The activity tracker had 2 main functionalities: (i) daily activity goal and (ii) inactivity alerts. The daily activity goal had 3 stages set by the tracker’s manufacturer. At baseline, the researcher responsible for the delivery of the intervention set the daily activity goal in Stage 1 for all the participants in the intervention group and later on, if the daily activity goal was frequently exceeded, the researcher suggested a higher daily activity goal to the intervention group member via e-mail or text message. Since the activity tracker had a built-in accelerometer, various kinds of activities contributed to the achieving of the daily activity goal: activities at higher intensities filled the daily goal faster than activities at lower intensities. The achievement of 100% of the daily activity goal at Stage 1 corresponded for instance to 2 h/day of walking, the Stage 2 to 3 h/day of walking, and the Stage 3 to 3.5 h/day of walking (35). The tracker provided daily information on how much activity had been accumulated and how much was still needed to reach the daily activity goal in a graphical form and messages such as “walk for 50 minutes” on the screen of the tracker.

The tracker prompted users to break up sedentary time by giving an inactivity alert as a vibration and the text “it’s time to move” appeared on the screen of the tracker if the person had been still without interruptions for 55 minutes.

The users also had a possibility to self-monitor their daily sedentary levels and amount of “inactivity stamps,” which the tracker recorded if the person did not start to move within 5 minutes after the inactivity alert, in their personal Polar Flow accounts either via a computer or a mobile phone app any time they wanted. The web-based Polar Flow program displayed overviews and summaries of the sedentary time on daily, weekly, and monthly base. Polar Flow also gave the users feedback on the daily activity goal attainment and, if the tracker had been worn sufficiently, a detailed feedback on health benefits based on daily physical activity and some information on the health benefits of reducing sedentary time such as “You spent quite a lot of time sitting down. You’ll see more health benefits if you reduce this.”

To summarize, the intervention was delivered solely by the activity tracker and web-based program, and the BCTs according to taxonomy of BCTTv1 (15) incorporated in the activity tracker and the web-based program to change specifically sedentary behavior included: prompts/cues [#7.1], self-monitoring of behavior [#2.3], feedback on behavior [#2.2], and information on health consequences [#5.1].

### Control Group

Participants randomized to the control group were requested to abstain from using of any type of activity trackers during the 12-month follow-up. As an incentive to continue in the study, the control group members were promised that they will receive a similar activity tracker used in the intervention after completing the 12-month follow-up measurements.

### Measures

#### Sedentary time

Accelerometer-based measurements were conducted at baseline, 3-, 6-, and 12-month time points. In this study, sedentary time was measured using a wrist-worn triaxial ActiGraph wGT3X-BT accelerometer, initialized to collect data at a sampling frequency of 80 Hz. Participants were instructed to wear the accelerometer on their nondominant wrist 8 consecutive days and nights at all times, including during water-based activities, but to remove the accelerometer while showering or having a sauna. They were also asked to report in-bed and out-of-bed times for each day they wore the accelerometer, along with times when they started and ended the measurement. The 12-month measurements were conducted at the same month with the baseline measurement (spring [44%], autumn [25%], and winter [31%] time).

The accelerometer data were analyzed according to a prespecified data reduction and analysis plan blinded for the allocation of the participants. Data from the accelerometers were downloaded using the ActiLife software (ActiGraph, Pensacola, FL) and processed using the open source R-package GGIR version 1.7-1 (R Foundation for Statistical Computing, Vienna, Austria, https://cran.r-project.org/) (36). The R-package GGIR script that we used is shown in the Supplementary File 3. The data were auto-calibrated and converted over 5-second epochs into Euclidean norm minus one vector magnitude units (36). Sleep time was estimated based on the method by van Hees et al (37), using both in-bed and out-of-bed times in the daily logs and algorithm of the GGIR package. Nonwear time was classified as part of the GGIR processing (38,39), using the standard deviation (*SD*) and value range of each accelerometer axis over 60-minute window centered on 15-minute time blocks. After the exclusion of sleep and nonwear, a minimum of 4 days with a minimum of 10 h/day measurement was required at each follow-up time point. This lead to exclusion of 1 follow-up measurement from 2 participants. Mean number of valid days across the follow-up was 7.5 (range 4–9). Sedentary time was defined using a previously proposed threshold of 30.0 mg (40). Daily prolonged sedentary time was defined as daily time spent in sustained sedentary bouts lasting ≥60 minutes, allowing breaks from sedentary behavior lasting less than 1 minute but requiring at least 90% of the bouts’ length to be below the sedentary threshold (41). The average prolonged sedentary bout length was also extracted from the data.

#### Participant characteristics

Demographic characteristics and the actual date of retirement were obtained by a web-based questionnaire before allocation into the intervention and control groups. Gender, date of birth, and occupational status were obtained from the pension institute’s (Keva) register. Occupational status was categorized based on the International Standard Classification of Occupations (ISCO) (42) into 3 groups according to the occupational titles by the last known occupation preceding retirement: managers and professionals (ISCO classes 1–2), associate professionals (ISCO classes 3–4), and manual and service workers (ISCO classes 5–9).

Body mass index at baseline was calculated based on measured height and weight, which was further categorized into under or normal weight (<25.0 kg/m^2^), overweight (25 to <30 kg/m^2^), and obese (≥30 kg/m^2^) (43). Only the control group included few underweight participants (*n* = 4). Smoking status was categorized as never/former and current and number of chronic diseases as none, 1, or ≥2 doctor-diagnosed chronic diseases (angina pectoris, claudication, myocardial infarction, or cerebrovascular disease, diabetes, osteoarthritis, osteoporosis, sciatica, fibromyalgia, rheumatoid arthritis, asthma, chronic bronchitis, depression, other mental disorder). Physical functioning was evaluated using the validated RAND-36 Health survey (identical with the Short Form SF-36) (44,45) as limitations in walking 2 km (no, yes). Physical activity level was determined based on self-reported weekly duration and intensity of leisure and commuting physical activity during the past year and categorized as “low,” that is, not meeting the physical activity recommendations (<14 metabolic equivalent hours per week) or “moderate to high,” that is meeting the physical activity recommendations (≥14 metabolic equivalent hours per week) (46).

#### Statistical analysis

Analyses were performed by the intention-to-treat principle so that all randomized participants were included in the analyses.

The characteristics of the study population at baseline are shown as percentages for the categorical variables and means and *SD*s for the continuous variables. Hierarchical linear mixed models were used to examine mean changes and differences in daily total sedentary time, daily prolonged sedentary time, and average prolonged sedentary bout length between the intervention and control group. The model included the intervention group as a between-factor, time as a within-factor, and the group by time interaction. Since the group * time interaction was significant for the accelerometer wake wear time (*p* = .04, partly due to differences in sleep time between the groups), all analyses were adjusted for the wake wear time to take into account the differences in the daily accelerometer wear time. Results are shown as mean estimates of daily total sedentary time and prolonged sedentary time and their 95% confidence intervals (CIs). As a post hoc analysis, we examined changes in daily total sedentary time, daily prolonged sedentary time, and average prolonged sedentary bout length from baseline to the 3-month time point and from baseline to the 6-month time point using hierarchical linear mixed models and adjusting for wake wear time. For the supplemental analyses, we stratified the study participants into tertiles according to the proportion prolonged sedentary time from daily total sedentary time (%) at baseline and examined the changes in prolonged sedentary time by the baseline proportion of prolonged sedentary time using hierarchical linear mixed models.

In the intervention group, the mean number of inactivity stamps was calculated per month and hierarchical linear mixed models were used to examine changes in the number of inactivity stamps per month across the intervention.

Sample size calculation was based on a previous RCT (47). Based on a power of 0.80 and 2-sided alpha of 0.05, 214 participants were required to detect a 12% unit difference (*SD* 31 (47)) between the intervention and the control groups in the primary outcome, accelerometer-measured wake-time physical activity at the 12-month time point. For sedentary time, no separate sample size calculations were conducted.

All statistical analyses were performed using the SAS statistical software, version 9.4 (SAS Institute, Inc., Cary, NC).

## Results

Baseline characteristics are presented in Table 1 and baseline daily sedentary time in Table 2. The mean age of the study population was 65.2 (*SD* 1.1) years and 83% of them were women. The control group participants were slightly more sedentary, but otherwise no differences were observed between the intervention and control groups at baseline. At the baseline accelerometer measurement, the mean total wear time of the accelerometer was 23 hours 51 minutes among the control group and 23 hours 45 minutes among the intervention group. Four participants from the intervention group and 1 participant from the control group were lost to follow-up, resulting in the dropout rate of 2% (Supplementary File 1). In the intervention group, the number of inactivity stamps per month increased during the intervention, particularly after the first 7 months of the intervention (*p* < .0001) (Supplementary File 4).

**Table 1.** Baseline Characteristics for the Intervention and the Control Groups

	Intervention Group (*n* = 117)	Control Group (*n* = 114)
Gender, *n* (%)		
Men	21 (18.0)	19 (16.7)
Women	96 (82.1)	95 (83.3)
Age, years, mean (*SD*)	65.2 (1.0)	65.2 (1.1)
Occupation, *n* (%)		
Managers/professionals	47 (40.2)	41 (36.0)
Associate professionals	35 (29.9)	28 (24.6)
Service/manual	35 (29.9)	45 (39.5)
BMI		
Under/normal weight	38 (32.5)	43 (37.7)
Overweight	43 (36.8)	45 (39.5)
Obese	36 (30.8)	26 (22.8)
Smoking, *n* (%)		
No	113 (96.6)	109 (96.5)
Yes	4 (3.4)	4 (3.5)
Number of chronic diseases, *n* (%)		
0	30 (25.6)	25 (21.9)
1	44 (37.6)	39 (34.2)
≥2	43 (36.8)	50 (43.9)
Limitations in walking 2 km		
No	109 (93.2)	106 (93.8)
Yes	8 (6.8)	7 (6.2)
Physical activity level		
Low	24 (20.5)	31 (27.2)
Moderate to high	93 (79.5)	83 (72.8)
Years from the retirement transition	1.2 (0.6)	1.1 (0.5)

*Notes*: BMI = body mass index; *SD* = standard deviation.

**Table 2.** Model-Based Means of Daily Total Sedentary Time and Prolonged Sedentary Time by Randomization Group (Intention-to-Treat Analysis)

		Intervention Group (*n* = 117)			Control Group (*n* = 114)			
	*n*	Mean	95% CI	*n*	Mean	95% CI	*p* Value Time Effect	*p* Value Time * Group Effect
Daily total sedentary time								
Baseline (min)	117	657	640 to 673	114	665	648 to 682		
Change at 3 months (min)	113	−11	−26 to 3	114	−17	−31 to −3		
Change at 6 months (min)	113	−24	−38 to −10	112	−13	−27 to 1		
Change at 12 months (min)	113	6	−8 to 20	112	4	−10 to 18	<.0001	.39
Daily prolonged sedentary time ≥60 min								
Baseline (min)	117	240	214 to 266	114	250	224 to 276		
Change at 3 months (min)	113	−9	−32 to 13	114	−3	−26 to 19		
Change at 6 months (min)	113	−28	−51 to −6	112	1	−21 to 24		
Change at 12 months (min)	113	13	−10 to 35	112	17	−5 to 40	.0042	.27

*Notes*: CI = confidence interval. Models adjusted for the wear time of the accelerometer during waking hours.

At baseline, the mean daily total sedentary time for the intervention group was 657 minutes (95% CI 640–673) of which 240 minutes (95% CI 214–266) was prolonged sedentary time (Table 2). Levels in the control group were slightly higher, 665 minutes (95% CI 648–682) and 250 minutes (95% CI 224–276), respectively (Table 2).

**Figure 1.**
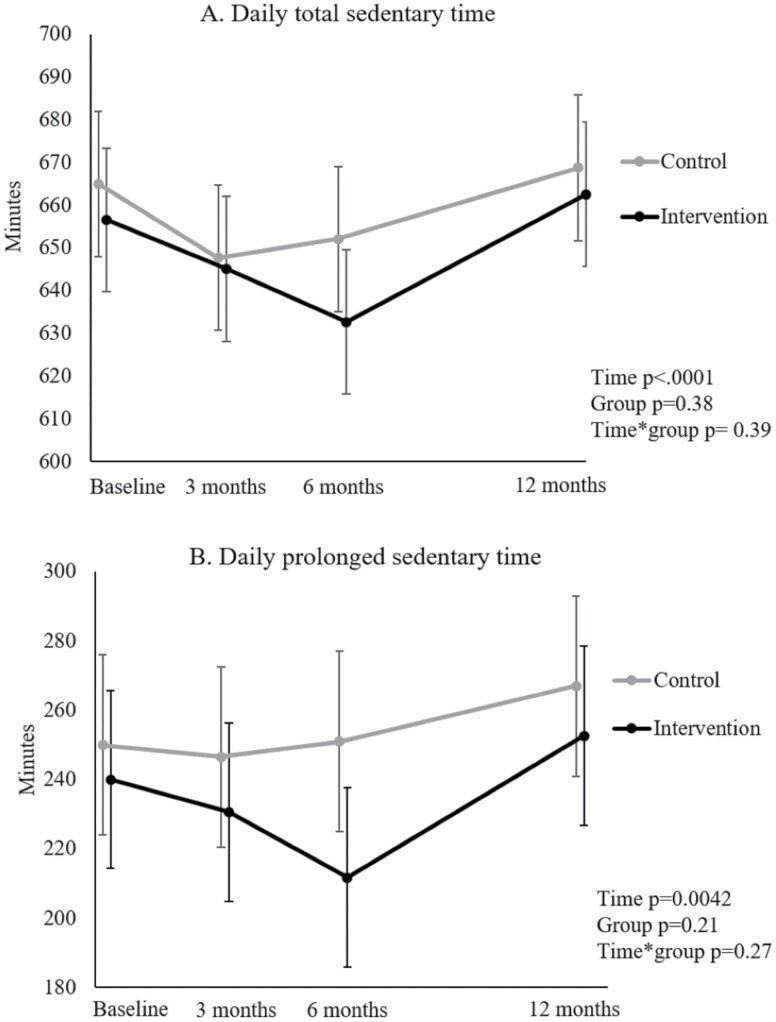
Changes in (A) daily total sedentary time and (B) daily prolonged sedentary time during the follow-up.

Figure 1 illustrates the changes in daily total and prolonged sedentary time across the 12-month intervention for the intervention and the control groups. Daily total sedentary time and prolonged sedentary time decreased during the first 6 months, followed by an increase during the last 6 months close to the baseline levels in both the intervention and the control group. Thus, the intervention had no effect either on daily total sedentary time (time * group interaction *p* = .39) nor on prolonged sedentary time (time * group interaction *p* = .27) over the 12-month intervention (Table 2).

We also examined changes in the average prolonged sedentary bout length across the 12-month intervention (Supplementary File 5). At baseline, the average prolonged sedentary bout length was 105 minutes (95% CI 100–111) in the intervention and 108 minutes (95% CI 103–114) in the control group and it remained relatively stable in both groups over time. No difference was observed between the intervention and control group over the 12 months (time * group interaction *p* = .88).

As a post hoc analysis, we examined 3-month and 6-month effects of the intervention. From baseline to 3 months, both the intervention and control groups showed a tendency to decrease daily total sedentary time, with no marked differences in changes between the groups (mean difference in changes −6 minutes, 95% CI −26 to 14). No significant changes were observed in prolonged sedentary time over the first 3 months. From baseline to 6 months, the intervention group’s daily total sedentary time decreased by 24 minutes (95% CI −38 to −10), but this change did not differ from that among the controls (−13 minutes, 95% CI −27 to 1, mean difference 11 minutes, 95% CI −9 to 31). The intervention group’s prolonged sedentary time decreased by 28 minutes (95% CI −51 to −6), while no changes were observed in the control group (1 minute, 95% CI −21 to 24), but the difference between the changes did not reach statistical significance, the mean difference in the changes being 29 minutes (95% CI −2 to 61). Among the intervention group, the average prolonged sedentary bout length decreased by 9 minutes (95% CI −15 to −2), but this change did not differ from that among the controls (mean difference between the changes 3 minutes, 95% CI −7 to 12).

The reduction in prolonged sedentary time from baseline to the 6-month time point was highest among those with the highest baseline proportion of prolonged sedentary time (Supplementary File 6). Those individuals with the highest proportion of prolonged sedentary time in the intervention group showed a gradual reduction of 120 minutes (95% CI −166 to −73) of prolonged sedentary time, but this change did not differ from that among the controls (−77 minutes, 95% CI −124 to −31, *p* value .20).

## Discussion

This study evaluated the effect of a 12-month consumer-based activity tracker intervention on daily total sedentary time and prolonged sedentary time among recent retirees. Our findings showed that the activity tracker that encouraged users to reach their daily physical activity goals and reminded to break up long uninterrupted inactivity periods was insufficient to elicit changes in daily total or prolonged sedentary time over the 12 months.

The REACT trial provides novel knowledge of the long-term effect of an activity tracker that was harnessed to deliver several BCTs targeting both sedentary behavior and physical activity. Previous activity tracker-based trials have been rather short-term (20,21) and utilized only self-monitoring of sedentary behavior (20) or physical activity (21,22) as the sole BCT to change sedentary behavior. We observed some indication of decreased daily total sedentary time, prolonged sedentary time, and average prolonged sedentary bout length at the 6-month time point, especially among the most sedentary study participants, but the differences between the intervention and control group were not statistically significant. The number of inactivity stamps increased during the intervention, which may indicate that some participants may have experienced initial interest in the use of an activity tracker that nevertheless attenuated toward the end of the intervention (48). These findings seem to correspond to recent qualitative findings among older adults, suggesting that activity trackers increase self-awareness but do not necessarily increase internal motivation to change health behaviors in the long-term (49,50). Therefore, some additional intervention components may be needed to maintain the initial interest in the activity tracker use.

The REACT trial is the first trial targeted to the time window immediately after transition to retirement (14,20–22), which has shown to induce unfavorable changes in sedentary behavior (9,10,12). Our findings showed that although an activity tracker was seen as a promising tool to deliver several evidence-based BCTs (20,30) and to be easily implemented in the nonoccupational contexts, it was insufficient to overcome the increasing trend of sedentary behavior after retirement. Interventions targeting certain sedentary activities, that is, watching TV, using a computer rather than sedentary time in general have been suggested, because people primarily engage in these activities for different reasons and sitting posture is only subservient to these activities (51). Inactivity alerts may be perceived as disruptive in contexts that require concentration, such as computer use, reading (52), or cultural events. Thus, further examinations of effective context-specific methods, especially targeted to reduce watching TV among retirees may be warranted.

Our study aimed to evaluate a consumer-based activity tracker as a stand-alone intervention component to reduce sedentary time; thus, the intervention relied on the BCTs incorporated into the activity tracker and the web-based program. The activity tracker and program included prompts, self-monitoring, feedback, and information on health benefits to change sedentary behavior, based on the constructs of the Social Cognitive Theory (24,25) and Health Belief Model (27). All other BCTs except for prompts were delivered through the program. Given that the participants were not requested to follow the information provided by the web-based program, it is not known how many of them actually utilized it. Among those who did, self-monitoring and information on activity benefits could have increased their awareness of the health consequences of sedentary behavior (23), and strengthened their responsiveness to the inactivity alerts (27).

Previous multicomponent interventions have combined activity tracker-delivered inactivity alerts and self-monitoring of physical activity with counseling emphasizing the health consequences of sedentary behavior, sedentary behavior-specific goal-setting, and identification of strategies to reduce sedentary time, which have resulted in nonsignificant or modest reductions in sedentary time (about 20–25 min/day) up to 6 months (17,18,53–55). A recent 3-month RCT among obese older adults (*n* = 60, mean age 68, the United States) reported 58 minutes greater reduction in daily sedentary time among the intervention group than among the controls, with a combination of activity tracker-delivered inactivity alerts every 15 minutes, identification of strategies to reduce sedentary time, and sedentary behavior-specific goal-setting (19). Thus, reflecting our findings, in addition to activity tracker-delivered prompts and self-monitoring of sedentary behavior, additional theory-based BCTs such as sedentary behavior-specific goal-setting, problem-solving, habit formation, and/or social support may be needed to achieve more robust, and possibly long-term reductions in sedentary time (15,25,27,56). Regarding inactivity alerts, being able to set more frequent inactivity alerts and individual goal-setting depending on the individual sedentary patterns could be more effective in reducing sedentary time. Also further efforts to combine the effective techniques targeted to sedentary time with the effective strategies to change physical activity at all intensities should be elucidated in future interventions.

Our study has several strengths. The participants were recruited according to their retirement date which enabled us to target the intervention to the time window immediately after retirement transition. We used accelerometer-based sedentary estimates as an outcome and conducted a long-term intervention with 4 measurement points, enabling us to evaluate accurately 3-month, 6-month, and 12-month effects of the intervention on both total daily sedentary time and prolonged sedentary time. The adherence to the intervention was excellent as the dropout rate was only 2%. Analyses were performed by the intention-to-treat principle so that all randomized participants were included in the analyses.

Our study has some limitations that should be noted. Baseline and follow-up measurements could have encouraged also the control group members to increase their physical activity and thus, simultaneously decrease sedentary time. The possibility that the control group participants utilized some type of an activity tracker during the follow-up cannot be ruled out, although the control group were requested to abstain from the use of any type of activity trackers. Seasonal variation in physical activity may also have affected the results. However, both the intervention and control group, assessed in 5 groups, started their 12-month follow-up in the same seasons: spring, winter, and autumn. Noteworthy is the fact that the wrist-worn activity tracker gives an inactivity alert when the user has been *still* but not necessarily sitting or lying down for 55 minutes. Validity of the Polar Loop 2 in estimating sedentary time has not yet been assessed, but modest correlation between sedentary time estimates from other Polar model, Polar M430 and a hip-worn triaxial ActiGraph has been reported (57). As a methodological limitation, using the GGIR package in processing data from wrist-worn accelerometers may overestimate absolute levels of daily sedentary time when compared to position-sensitive thigh-worn accelerometers (58). Consequently, the absolute levels of daily sedentary time were relatively high (about 11 h/day), but these levels corresponded to the levels observed in another study utilizing the GGIR package in processing data from wrist-worn accelerometers among participants from the same age group (59). However, instead of absolute levels, we studied changes in sedentary time, which has been shown to be reliably captured with wrist-worn accelerometers (58).

Our results may not be generalized to general population because our study population included former public sector workers in Finland of whom majority (78%) are women (60). However, there is high diversity of occupations among the public sector workers, thus our findings can be generalized to people retiring from a variety of occupations.

## Conclusions

The use of a consumer-based activity tracker with inactivity alerts did not reduce sedentary time over 12 months among a general study population of recent retirees. Alternative approaches are needed to examine how to induce long-term reductions in sedentary time among retirees.

## Supplementary Material

glab107_suppl_Supplementary_File_1

glab107_suppl_Supplementary_File_2

glab107_suppl_Supplementary_File_3

glab107_suppl_Supplementary_File_4

glab107_suppl_Supplementary_File_5

glab107_suppl_Supplementary_File_6

## Data Availability

Anonymized partial data sets of the REACT trial are available by application with bona fide researchers with an established scientific record and bona fide organizations.

## Declarations

Ethics approval and consent to participate: The REACT study is conducted in line with the Declaration of Helsinki, and has been approved by the Ethics Committee of Hospital District of Southwest Finland (107/1801/2017). All participants provided their full written consent to participate in the study.

Consent for publication: Not applicable.
