# Supplementary figures and images for: The Effect of a Consumer-Based Activity Tracker Intervention on Accelerometer-Measured Sedentary Time Among Retirees: A Randomized Controlled REACT Trial
Source: J Gerontol A Biol Sci Med Sci. 2021 Apr 11;77(3):579–87. doi: 10.1093/gerona/glab107 (PMC8893187; doi:10.1093/gerona/glab107)

**Supplemental file 1.** Flow Diagram.

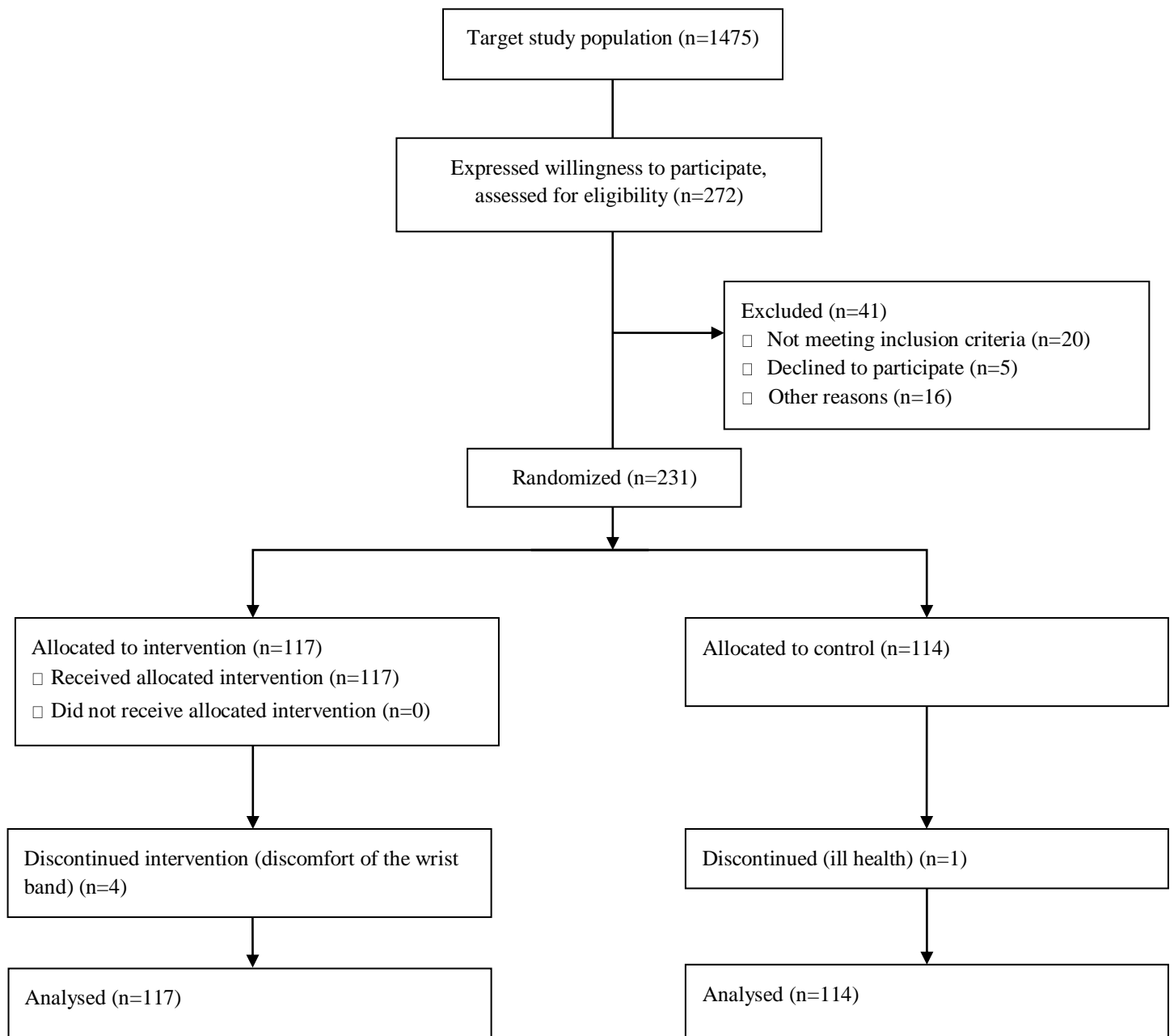

Supplement: glab107_suppl_Supplementary_File_1 [file glab107_suppl_supplementary_file_1.pdf]

## Supplemental file 6. Changes in daily prolonged sedentary time by tertiles

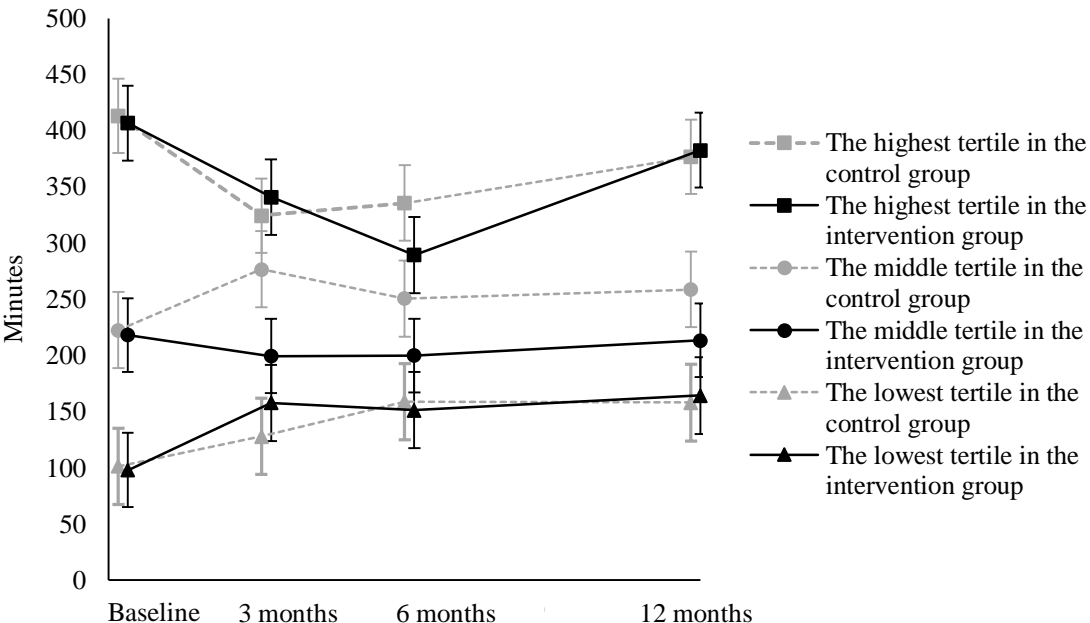

Supplement: glab107_suppl_Supplementary_File_6 [file glab107_suppl_supplementary_file_6.pdf]
