# Supplementary material for: The Effect of a Consumer-Based Activity Tracker Intervention on Accelerometer-Measured Sedentary Time Among Retirees: A Randomized Controlled REACT Trial
Source: J Gerontol A Biol Sci Med Sci. 2021 Apr 11;77(3):579–87. doi: 10.1093/gerona/glab107 (PMC8893187; doi:10.1093/gerona/glab107)
Supplement: glab107_suppl_Supplementary_File_3 [file glab107_suppl_supplementary_file_3.pdf]

Supplemental file 3 The GGIR script.

```
library(GGIR)


f0=1

f1=231


g.shell.GGIR(#=======================================

        mode=c(1,2,3,4,5),

        datadir="/wrk/data",

        outputdir="/wrk/results",

        f0=f0, f1=f1,

        daylimit=FALSE,

        #-------------------------------

        # Part 1:

        #-------------------------------

        windowsizes = c(5, 900, 3600),

        desiredtz="Europe/Helsinki",

        do.enmo = TRUE,         do.anglez=TRUE,

        chunksize=1,            printsummary=TRUE,

        overwrite=TRUE,

        #-------------------------------

        # Part 2:

        #-------------------------------

        strategy = 3,

        ndayswindow=9,

        winhr = c(5),

        qwindow=c(0,24),
```

```
ilevels = c(seq(0,400,by=50),8000),
mvpathreshold =c(100.6),
bout.metric = 4,
epochvalues2csv=FALSE,
closedbout=FALSE,
do.imp = FALSE,
#-------------------------------
# Part 3:
#-------------------------------
# Key functions: Sleep detection
timethreshold= c(5),       anglethreshold=5,
ignorenonwear = TRUE,
desiredtz="Europe/Helsinki",
 #-------------------------------
 # Part 4:
 #-------------------------------
 excludefirstlast = FALSE,
 includenightcrit = 16,
 def.noc.sleep = c(),
 loglocation= c("/wrk/KL_log.csv"),
 outliers.only = TRUE,
 criterror = 4,
 relyonsleeplog = FALSE,
 sleeplogidnum = TRUE,
 colid=1,
 coln1=2,
 do.visual = TRUE,
```

```
nnights = 9,

#------------------------------

# Part 5:

# Key functions: Merging physical activity with sleep analyses

#------------------------------

threshold.lig = c(30), threshold.mod = c(100.6),  threshold.vig = c(428.8),

boutcriter = 0.8,      boutcriter.in = 0.9,     boutcriter.lig = 0.8,

boutcriter.mvpa = 0.8, boutdur.in = c(1,30,60), boutdur.lig = c(1,10),

boutdur.mvpa = c(1,10),   timewindow = c("WW"), save_ms5rawlevels= TRUE,

#----------------------------------

# Report generation

#------------------------------

# Key functions: Generating reports based on meta-data

do.report=c(2,4,5),

visualreport=TRUE,     dofirstpage = TRUE,

viewingwindow=1)
```
