# Supplementary material for: The Effect of a Consumer-Based Activity Tracker Intervention on Accelerometer-Measured Sedentary Time Among Retirees: A Randomized Controlled REACT Trial
Source: J Gerontol A Biol Sci Med Sci. 2021 Apr 11;77(3):579–87. doi: 10.1093/gerona/glab107 (PMC8893187; doi:10.1093/gerona/glab107)

Supplemental file 4. Mean number of inactivity stamps (with 95% confidence intervals) per month across the 12-month intervention (based on linear mixed model).

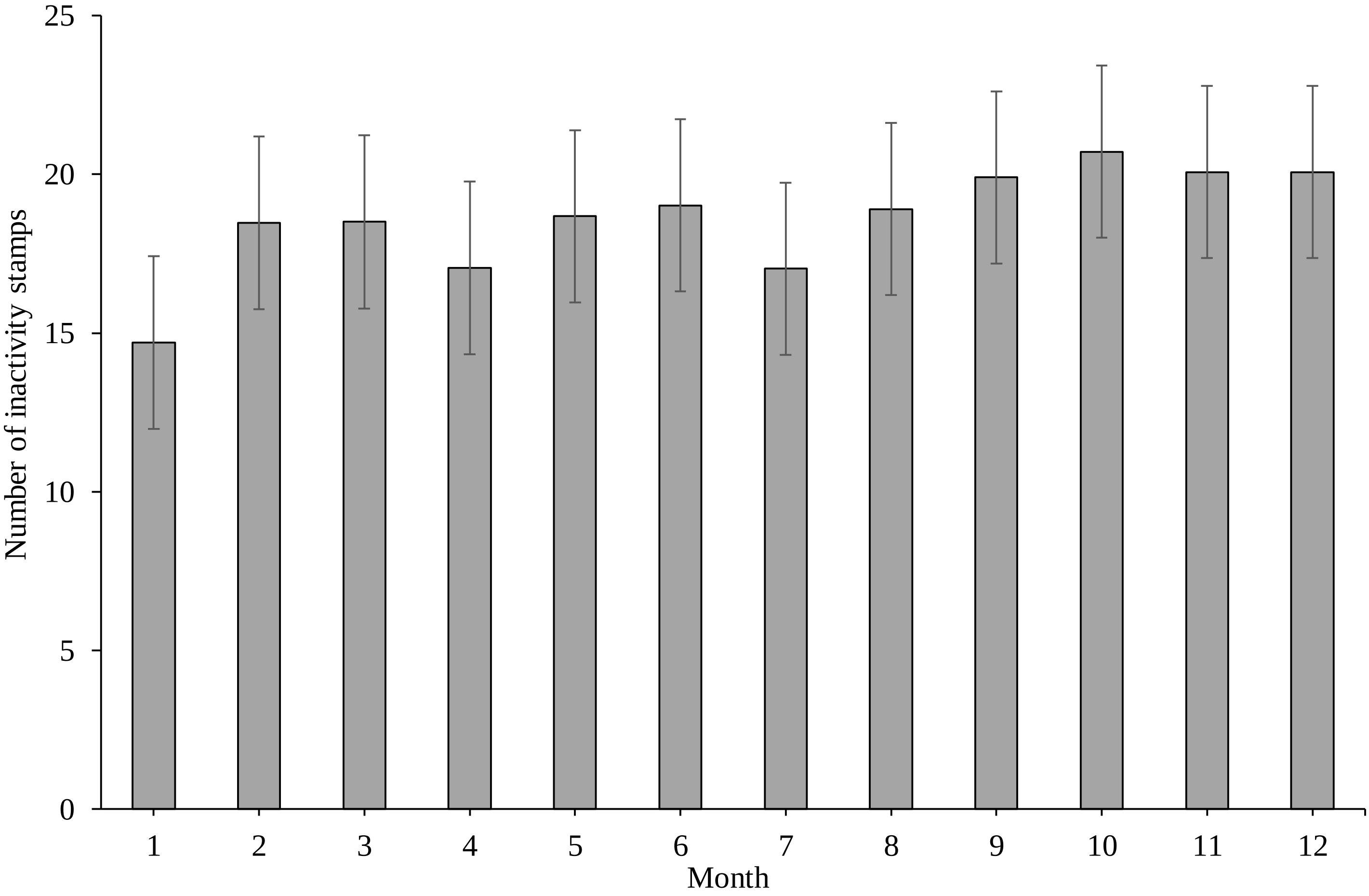

Supplement: glab107_suppl_Supplementary_File_4 [file glab107_suppl_supplementary_file_4.pdf]
