# Supplementary material for: The Effect of a Consumer-Based Activity Tracker Intervention on Accelerometer-Measured Sedentary Time Among Retirees: A Randomized Controlled REACT Trial
Source: J Gerontol A Biol Sci Med Sci. 2021 Apr 11;77(3):579–87. doi: 10.1093/gerona/glab107 (PMC8893187; doi:10.1093/gerona/glab107)
Supplement: glab107_suppl_Supplementary_File_5 [file glab107_suppl_supplementary_file_5.pdf]

Supplemental file 5. Model-based means of the prolonged sedentary bout length by randomization group (intention to treat analysis).

	Intervention group n=117			Control group n=114				
	n	Mean	95% CI	n	Mean	95% CI	P-value Time effect	P-value Time*group effect
Prolonged sedentary bout length								
Baseline (min)	117	105	100 to 111	114	108	103 to 114		
Change at 3 months (min)	113	-4	-10 to 3	114	-4	-11 to 2		
Change at 6 months (min)	113	-9	-15 to -2	112	-6	-12 to 1		
Change at 12 months (min)	113	-6	-12 to 1	112	-6	-13 to 1	0.020	0.88
